# Combinatorial metabolic engineering of *Bacillus subtilis* enables the efficient biosynthesis of isoquercitrin from quercetin

**DOI:** 10.1186/s12934-024-02390-5

**Published:** 2024-04-20

**Authors:** Tengfei Niu, Chaokang Huang, Rufeng Wang, Li Yang, Shujuan Zhao, Zhengtao Wang

**Affiliations:** 1 Institute of Chinese Materia Medica, Shanghai University of Traditional Chinese Medicine, Shanghai, 201203 China; 2 The SATCM Key Laboratory for New Resources & Quality Evaluation of Chinese Medicine, Institute of Chinese Materia Medica, Shanghai University of Traditional Chinese Medicine, Shanghai, 201203 China; 3 The MOE Key Laboratory for Standardization of Chinese Medicines and Shanghai Key Laboratory of Compound Chinese Medicines, Shanghai University of Traditional Chinese Medicine, Shanghai, 201203 China

**Keywords:** Isoquercitrin, UDP-glucose, Combinatorial metabolic engineering, Biotransformation, *Bacillus subtilis*

## Abstract

**Background:**

Isoquercitrin (quercetin-3-O-β-D-glucopyranoside) has exhibited promising therapeutic potentials as cardioprotective, anti-diabetic, anti-cancer, and anti-viral agents. However, its structural complexity and limited natural abundance make both bulk chemical synthesis and extraction from medical plants difficult. Microbial biotransformation through heterologous expression of glycosyltransferases offers a safe and sustainable route for its production. Despite several attempts reported in microbial hosts, the current production levels of isoquercitrin still lag behind industrial standards.

**Results:**

Herein, the heterologous expression of glycosyltransferase UGT78D2 gene in *Bacillus subtilis* 168 and reconstruction of UDP-glucose (UDP-Glc) synthesis pathway led to the synthesis of isoquercitrin from quercetin with titers of 0.37 g/L and 0.42 g/L, respectively. Subsequently, the quercetin catabolism blocked by disruption of a quercetin dioxygenase, three ring-cleavage dioxygenases, and seven oxidoreductases increased the isoquercitrin titer to 1.64 g/L. And the hydrolysis of isoquercitrin was eliminated by three β-glucosidase genes disruption, thereby affording 3.58 g/L isoquercitrin. Furthermore, UDP-Glc pool boosted by *pgi* (encoding glucose-6-phosphate isomerase) disruption increased the isoquercitrin titer to 10.6 g/L with the yield on quercetin of 72% and to 35.6 g/L with the yield on quercetin of 77.2% in a 1.3-L fermentor.

**Conclusion:**

The engineered *B. subtilis* strain developed here holds great potential for initiating the sustainable and large-scale industrial production of isoquercitrin. The strategies proposed in this study provides a reference to improve the production of other flavonoid glycosides by engineered *B. subtilis* cell factories.

**Supplementary Information:**

The online version contains supplementary material available at 10.1186/s12934-024-02390-5.

## Background

Isoquercitrin (quercetin-3-*O*-β-d-glucopyranoside), a major glycosidic product ubiquitously distributed in various medicinal herbs, has drawn increasing attention due to its multifaceted health benefits, including cardioprotective, anti-diabetic, anti-anaphylactic, anti-cancer, and anti-viral properties [1–4]. Therefore, isoquercitrin has recently emerged as a significant candidate for functional food supplementation and herbal medicine in the management of cardiovascular disorders, renal injury, and diabetes mellitus-related vascular complications [3, 5–7]. Currently, isoquercitrin has been extracted from various parts of plant, including flowers, leaves, fruits, stems, and seeds [1]. However, it is characterized by inefficiency, time-intensiveness, and laboriousness to isolate the isoquercitrin in a pure state for food and pharmaceutical industries due to its extremely low content in plant material [1, 8]. Alternatively, using microbes as biocatalysts offers a promising way to produce high-value compounds.

According to the previous reports, the hydrolysis of rutin using microorganisms has been utilized to produce isoquercitrin [8]. Several microorganisms have been documented for their ability to produce isoquercitrin from rutin hydrolysis, such as *Aspergillus niger*, *Bacillus litoralis* C44, and *Chloroflexus aurantiacus* [8–10]. Furthermore, an alkali-tolerant and thermo-stable α-L-rhamnosidase derived from *Aspergillus terreus*, heterologously expressed in *Komagataella phaffii (Pichia pastoris)*, demonstrated the ability to efficiently convert rutin into isoquercitrin in significantly enhanced productivity [11, 12]. The purified extracellular α-L-rhamnosidase efficiently catalyzed the transformation of isoquercitrin from rutin and this procedure yielded the product of isoquercitrin up to 300 g/L [12]. Although the higher productivity of isoquercitrin had been achieved from rutin hydrolysis, the production of isoquercitrin through glycosylation of quercetin is yet to be deeply investigated.

Recently, direct biotransformation of quercetin using microorganisms harboring glycosyltransferases to produce isoquercitrin is the preferred approach [13, 14]. It was reported that a yield of 3.9 g/L isoquercitrin was achieved from quercetin by disrupting the *pgi* gene in *Escherichia coli* MG1655 expressing UGT73B3 [13]. Furthermore, through coupling the sucrose synthase GmSUS from *Glycine max* and the glycosyltransferase UGT78D2 in *E. coli* BL21(DE3), isoquercitrin was produced at titers of 3.83 g/L with the yield on quercetin of 94.3% in a fed-batch manner [15, 16]. However, the production of isoquercitrin from quercetin was low by *E. coli* expressing glycosyltransferases. In addition, the isoquercitrin produced by *E. coli* does not achieve generally recognized as safe (GRAS) status, thereby limiting its application in medical and pharmaceutical industry. Thus, there have attempts to select other microorganism, such as *Bacillus subtilis* has been explored as potential hosts for large-scale industrial production of pharmaceutical biochemicals due to their GRAS status. Numerous studies have showcased the utilization of *B. subtilis* as a cell factory for the synthesis of vitamins and nutraceuticals [17, 18]. Recently, *B. subtilis* has emerged as a promising host for producing natural products, such as amorphadiene, taxadiene, carotenoids, squalene, α-monoglucosyl hesperidin, and tetramethylpyrazine [19–23].

**Fig. 1:**
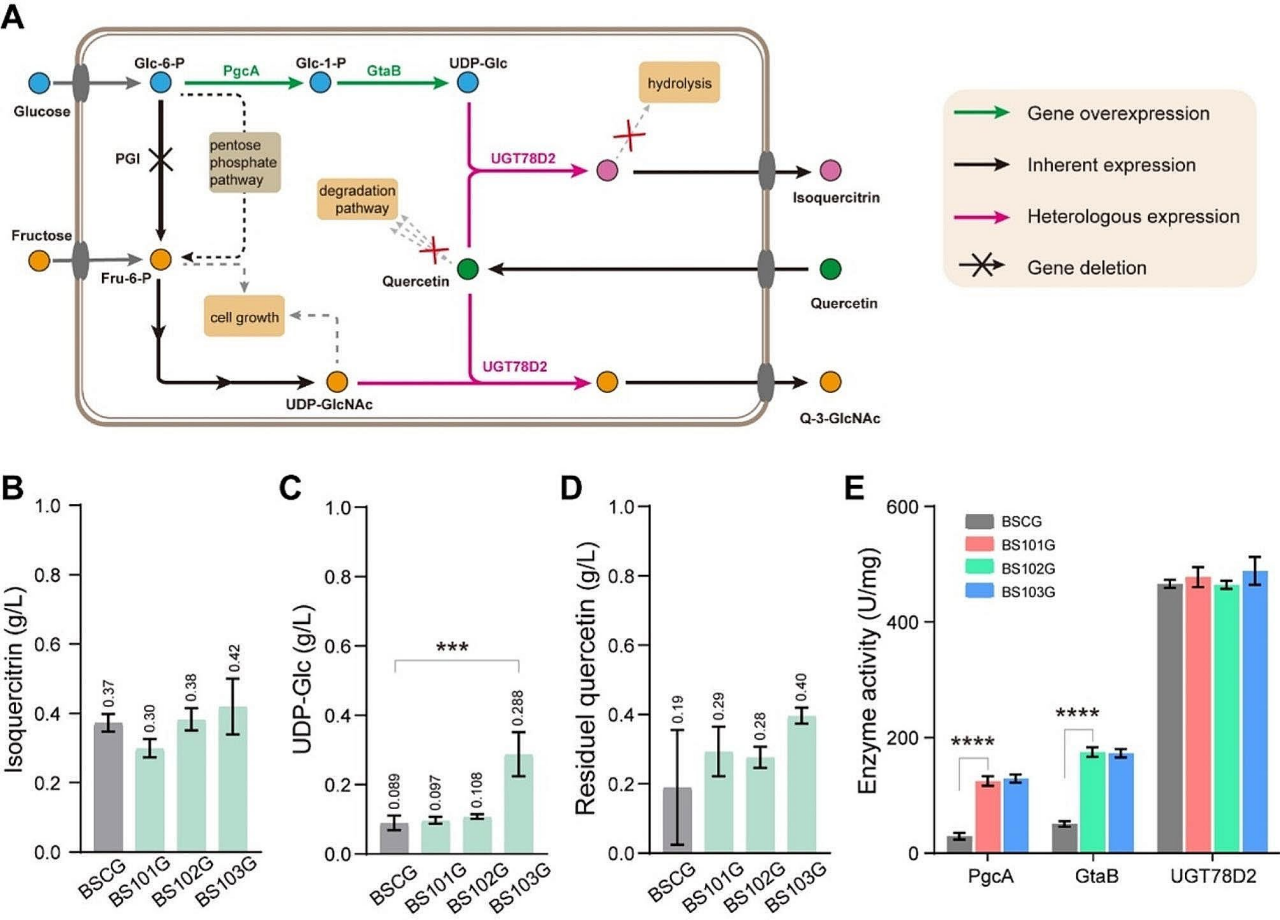
Combinatorial metabolic engineering of *Bacillus subtilis* for the synthesis of quercetin glycosides. (**A**) Biosynthetic pathway of quercetin glycosides. Glc-6-P, glucose-6-phosphate; Glc-1-P, glucose-1-phosphate; UDP-Glc, UDP-glucose; Fru-6-P, fructose-6-phosphate; UDP-GlcNAc, UDP-acetylglucosamine; Isoquercitrin, quercetin-3-O-glucoside; Q-3-GlcNAc, quercetin-3-acetylglucosamine; PgcA, glucose phosphate mutase; GtaB, glucose-1-phosphate uridyltransferase; PGI, glucose-6-phosphate isomerase; UGT78D2, UDP-glucosyltransferase. (**B**) Influence of engineering UDP-Glc synthesis pathway on isoquercitrin synthesis. (**C**) Influence of engineering UDP-Glc synthesis pathway on intracellular UDP-Glc concentration. (**D**) The concentration of residual quercetin. (**E**) Activity profiles in crude lysates of *B. subtilis* recombinants BSCG (initial host), BS101G (*pgcA* overexpressed under the control of strong constitutive promoter *P*_*43*_), BS102G (*gtaB* overexpressed under the control of strong constitutive promoter *P*_*srfA*_), BS103G (*pgcA* and *gtaB* overexpressed under the control of strong constitutive promoter *P*_*43*_ and *P*_*srfA*_, respectively)

Sugar nucleotides, such as UDP-glucose (UDP-Glc), UDP-xylose, UDP-acetylglucosamine, serve as activated sugars in many enzymatic glycosylation reactions catalyzed by specific glycosyltransferases, which are involved in the synthesis of glycosides in medical plants. In recent, the glycosyltransferases were widely used for the biosynthesis of glycosides in microorganisms [24]. In the biosynthesis of glycosides, a substantial quantity of costly UDP-sugars must be expended. To overcome this limitation, one alternative is to use microorganisms capable of regenerating UDP-sugar in vivo. In general, bacteria have the inherent biosynthesis pathways to make nucleotide sugar such as UDP-Glc and UDP-acetylglucosamine for cell wall biosynthesis and membrane modification (Fig. 1A) [14]. Hence, sufficient supply of UDP-sugar by engineering microorganisms is vital for production of glycosides. Recent studies have shown that enhancing the expression of UDP-sugar biosynthesis pathway genes in the host cell can effectively increase UDP-sugar supply [25, 26]. Therefore, UDP-glucose biosynthesis pathway needs to be engineered rationally to increase UDP-glucose supply in *B. subtilis* 168 expressing glycosyltransferase UGT78D2 for the synthesis of isoquercitrin (quercetin-3-*O*-glucoside).

Herein, a *B. subtilis* 168 strain was engineered to efficiently convert quercetin into isoquercitrin by optimizing UDP-glucose (UDP-Glc) supply and enhancing the glycosylation of quercetin. By reconstructing the synthesis pathway of UDP-Glc and redistributing carbon flow towards UDP-Glc synthesis through *pgi* disruption, the glycosyl donor UDP-Glc pool was significantly boosted. Furthermore, the quercetin catabolism was successfully blocked by deleting key genes involved in its degradation, including a quercetin dioxygenase gene (*yxaG*), three ring-cleavage dioxygenase genes (*ydfO*, *mhqA*, and *yodE*), and seven oxidoreductase genes (*yhjG*, *yetG*, *yetO*, *catE*, *moxC*, *ycnE*, and *yoaI*). In addition, three β-glucosidases (BglC, BglH, and BglA) involved in the deglycosylation of isoquercitrin were also eliminated to enhance the accumulation of isoquercitrin. Consequently, the engineered *B. subtilis* strain capable of improved UDP-Glc supply, eliminated quercetin degradation, and reduced glycoside deglycosylation is suitable for flavonoid glycosides biosynthesis, which increase isoquercitrin titers to 35.6 g/L with the yield on quercetin of 77.2% in a 1.3-L fermentor. Overall, we expect that the engineered *B. subtilis* strain developed here holds great potential for initiating the sustainable and large-scale industrial production of flavonoid glycosides.

## Materials and methods

### Strains and reagents

*B. subtilis* 168 was used as the initial host for genetic manipulation and strain construction. Gene fragments were amplified using PrimeSTAR Max DNA polymerase with the chromosomal DNA of *B. subtilis* as the template. The codon-optimized gene UGT78D2 (NCBI Accession Number: Q9LFJ8.1) from *Arabidopsis thaliana* was synthesized by Songon, and its gene sequence is listed in Table S1. The sequence of UGT78D2 was inserted into the pP_*43*_NMK vector using the *KpnI* and *XbaI* restriction sites, yielding the recombinant plasmid p*P*_*43*_-UGT78D2. The strains and plasmids constructed in this work are listed in the Table S2, and primers used in this work are listed in the Table S3. PrimeSTAR Max DNA polymerase was purchased from Takara. DNA gel purification kit and plasmid extraction kit were purchased from Generay. Quercetin and isoquercitrin were purchased from Nakeli.

### Genetic engineering

The gene deletion and integration were accomplished in *B. subtilis* using marker-free knockout approach [27]. The front homology region, back homology region flanking the genetic target and *lox77*-*zeo*-*lox66* cassette were amplified using the chromosomal DNA of *B. subtilis* and p7Z6 as the template, respectively. Deletion cassettes were constructed through overlapping extension polymerase chain reaction (PCR). The resulting PCR products were transformed into *B. subtilis* cells according to the competent cell preparation method [28]. For gene deletion, roughly 700 bp sequences homologous to the front and back regions of the chromosomal target were needed for homologous recombination. Zeocin at a concentration of 20 µg/mL and ampicillin at a concentration of 100 µg/mL were used for selecting strains with removed resistance marker cassettes and cured temperature-sensitive vectors, respectively. For gene overexpression, the specific promoter embedded within the deletion cassette was integrated into the designated chromosomal loci to provide a stable and high-level expression of target gene. All gene manipulation were verified by analysis of PCR products using agarose gel electrophoresis (Fig. S1).

### Strain cultivation

All *B. subtilis* strains and *E. coli* were cultured in Luria-Bertani Broth (10 g/L tryptone, 5 g/L yeast extract, and 10 g/L NaCl) at 37 ^o^C. The fermentation medium contained the following components (g/L): tryptone 6.0, yeast extract 12.0, (NH_4_)_2_SO_4_ 6.0, K_2_HPO_4_·3H_2_O 12.5, KH_2_PO_4_ 2.5, MgSO_4_·7H_2_O 1.0, and glucose 60.0. To investigate the quercetin degradation, 4 g/L quercetin was supplemented to the fermentation medium. For isoquercitrin biosynthesis, 6 g/L, 8 g/L, 10 g/L, 12 g/L quercetin were used to supplement adequate substrate. After incubation at 37 ^o^C for 12 h, quercetin was added into the culture for the quercetin glycoside biosynthesis.

### Fed-batch fermentation

Fed-batch fermentations were conducted in a 1.3 L bioreactor (T&J Bio-engineering Co., Ltd, China) with a working volume of 0.7 L. The fed-batch fermentation medium comprised 3% glucose and fructose, 0.6% tryptone, 1.2% yeast extract, 0.6% (NH_4_)_2_SO_4_, 1.25%K_2_HPO_4_·3H_2_O, 0.25% KH_2_PO_4_, and 0.1%MgSO_4_·7H_2_O. First, a colony was inoculated in the shake flask containing 30 mL LB to prepare the seed culture. Second, after 12 h of culture, seed liquid was inoculated into the 1.3 L bioreactor with 10% of the inoculation amount. The aeration rate was kept at 1.5 vvm, and dissolved oxygen (DO) was kept at a saturation rate of > 30% through the stirring rate (600–900 rpm). The fermentation temperature was maintained at 37 ^o^C, and the pH was kept at 7.0 through addition of 10% H_3_PO_4_ and 30% NH_3_·H_2_O. Subsequently, glucose concentration was kept at 20 g/L by feeding 600 g/L glucose (600 g/L glucose and 200 g/L fructose) at adjusted feeding rates for the cell growth. At 24 and 48 h of culture, 10–15 g/L quercetin were supplied for the biosynthesis of quercetin glycoside, respectively.

### Metabolites extraction and quantification

The concentrations of quercetin and isoquercitrin were determined using an Agilent 1260 HPLC system equipped with a UV detector at 254 nm and a 4.6 × 240 mm Zorbax SB-C18 (4.6 × 250 mm, 5 μm, Agilent) column. Acetonitrile (A) and water (containing 0.5% boric acid) (B) served as the mobile phases at a flow rate of 1 mL/min and 30 ^o^C. The concentrations of fructose and glucose were determined by HPLC (Agilent 1260, Bio-Rad, Hercules, CA; HPX-87 H column) equipped with refractive index detector using 5 mM H_2_SO_4_ as the mobile phase at a flow rate of 0.5 mL/min and 30 °C. Sample injection volume was 10 µL. To determine the intracellular UDP-Glc concentration, the culture was harvested at a specified time point, centrifugated and resuspended in cold PBS buffer (100 mM, pH7.2) to an OD_600_ of 200. Then, 100 µL of the culture with an OD_600_ of 200 was mixed with 400 µL of pre-cooled acetonitrile: methanol mixture (50:50, *v/v*) to incubate at -20 ^o^C for 30 min. Afterwards, cell debris was removed by centrifugation and the supernatant was collected for further analysis. Intracellular UDP-Glc analysis was performed on an Agilent 1260 system using a SeQuant ZIC®-cHILIC (4.6 × 250 mm, 3 μm, Supelco) column, maintained at 35 ^o^C with UV detection at 254 nm. The mobile phases consisted of acetonitrile (A) and water (B) containing 25 mM NH_4_Ac at pH6.8 with a flow rate of 0.5 mL/min. The elution gradients were as follows: 20–35% B (0–5 min), 35–50% B (5–15 min), 50–60% B (15–16 min), 60% B (16–18 min), 60 − 10% B (18–19 min), and 10–20% B (19–20 min). The intracellular concentration of UDP-Glc was calculated using 1 mL culture with an OD_600_ of 1 and a total cell volume of 0.56 µL.

### Liquid chromatography-mass spectrometry analysis

Liquid chromatography-mass spectrometry (LC-MS) analysis was performed using An ACQUITY UPLC® HSS T3 C18 column (2.1 × 100 mm, 1.8 μm, Waters) on an AB Sciex Q-TOF 5600 + LC/MS instrument equipped with an electrospray ionization source. The operation parameters were set as follows: ion spray voltage, -4.5 kV; cone voltage, 65 V; fragmentor voltage, 175 V; nebulizer pressure, 50 psi; capillary temperature, 500 ^o^C; desolvation gas flow, 5 mL/min; cone gas flow, 5 mL/min; and m/z range, 100–1000. Gradient elution was performed with (A) acetonitrile and (B) water (containing 0.1% formic acid) as the mobile phases at a flow rate of 0.4 mL/min. The elution gradients were as follows: 10% B (0–2 min), 10–35% B (2–10 min), 35–42% B (10–14 min), 42–95% B (14–24 min), 95% B (24–30 min).

### Enzyme activity assays

For enzyme activity assays, cells were harvested at given time and disrupted by high pressure homogenization crushing. After centrifugation at 10,000×g for 20 min, the supernatant was used for activity assays. In the UGT78D2 activity assay, 1 mg/mL crude extract was incubated in a 200 µL reaction mixture containing 10 mM quercetin, 10 mM UDP-Glc, 2 mM MgSO_4_·7H_2_O, and 100 mM potassium phosphate buffer (pH7.2) at 37 ^o^C for 30 min. Glucose phosphate mutase (PgcA) activity was directly measured by monitoring the transformation of glucose-6-phosphate to glucose-1-phosphate at 37 ^o^C in 100 mM potassium phosphate buffer (pH7.2). Glucose-1-phosphate uridyltransferase (GtaB) activities were measured in tubes in 200 µL reaction volumes. The reaction contained 100 mM potassium phosphate buffer (pH7.2), 10 mM glucose-1-phosphate, 10 mM UTP, 10 mg/L MgCl_2_, and 1 mg/mL crude enzyme. The reactions were terminated by adding 200 µL methanol and followed by centrifugation for 30 min. The supernatant was then used for HPLC analysis. One unit of enzyme was defined as the picomoles of product formed from the corresponding substrate per milligrams of protein in 1 min under the assay conditions.

### Statistical analysis

Data are mean ± standard deviation for *n* = 3 biological replicates. *P < 0.05, **P < 0.01, ***P < 0.001, ****P < 0.0001 as determined by *t*-tests.

## Results

### Engineering UDP-glucose synthesis pathway to improve isoquercitrin production

To enhance the carbon flux towards UDP-glucose synthesis pathway, these two promoters *P*_*43*_ and *P*_*srfA*_ were separately integrated into the upstream of *pgcA* (encoding glucose phosphate mutase) and *gtaB* (encoding glucose-1-phosphate uridyltransferase) in BSCG to increase the expression of either *pgcA* and *gtaB*, generating the strains BS101G and BS102G, respectively. Additionally, both the promoters *P*_*43*_ and *P*_*srfA*_ were successively integrated into the upstream of *pgcA* or *gtaB* in BSCG to generate the strain BS103G. After fermentation for 48 h, UGT78D2 overexpressing strain BSCG gave the low isoquercitrin titer of 0.37 g/L with a yield on quercetin of 9.2% in shake flask (Fig. 1B). In recombinant strains BS101G, BS102G, and BS103G, the isoquercitrin titers were determined as 0.30, 0.38, and 0.42 g/L, respectively (Fig. 1B). Compared with BSCG, the UDP-Glc synthesis pathway engineered strains BS101G, BS102G, and BS103G showed no increase in isoquercitrin titer. Therefore, the concentrations of intracellular UDP-Glc were further analyzed. These results showed that there was no difference between UDP-Glc synthesis pathway engineered strains BS101G, BS102G, and the control BSCG in intracellular UDP-Glc concentration (Fig. 1C). However, simultaneous overexpression of both *pgcA* and *gtaB* gave the intracellular UDP-Glc titer of 0.288 g/L, which was 223.6% higher than that accumulated in the control stain BSCG (Fig. 1C). The findings indicated that the enhanced UDP-Glc levels in BS103G has no effect on the production of isoquercitrin. Therefore, we further investigated the changes in quercetin titers. After incubation for 48 h, HPLC analysis revealed up to 90% of quercetin was depleted (Fig. 1D). The biosynthesis of isoquercitrin should be further investigated.

The enzyme activities of PgcA, GtaB, and UGT78D2 were further analyzed. The specific PgcA activities were increased to 124.9 U/mg (BS101G) and 129.3 U/mg (BS103G), which were 4.25- and 4.40-fold the level in BSCG (Fig. 1E). The specific GtaB activities were increased to 175.1 U/mg (BS102G) and 173.0 U/mg (BS103G), which were 3.45- and 3.41-fold the level in BSCG (Fig. 1E). In recombinant strains BSCG, BS101G, BS102G, and BS103G, the specific UGT78D2 activities were 466.4, 478.2, 464.8, and 488.8 U/mg, respectively.

**Fig. 2:**
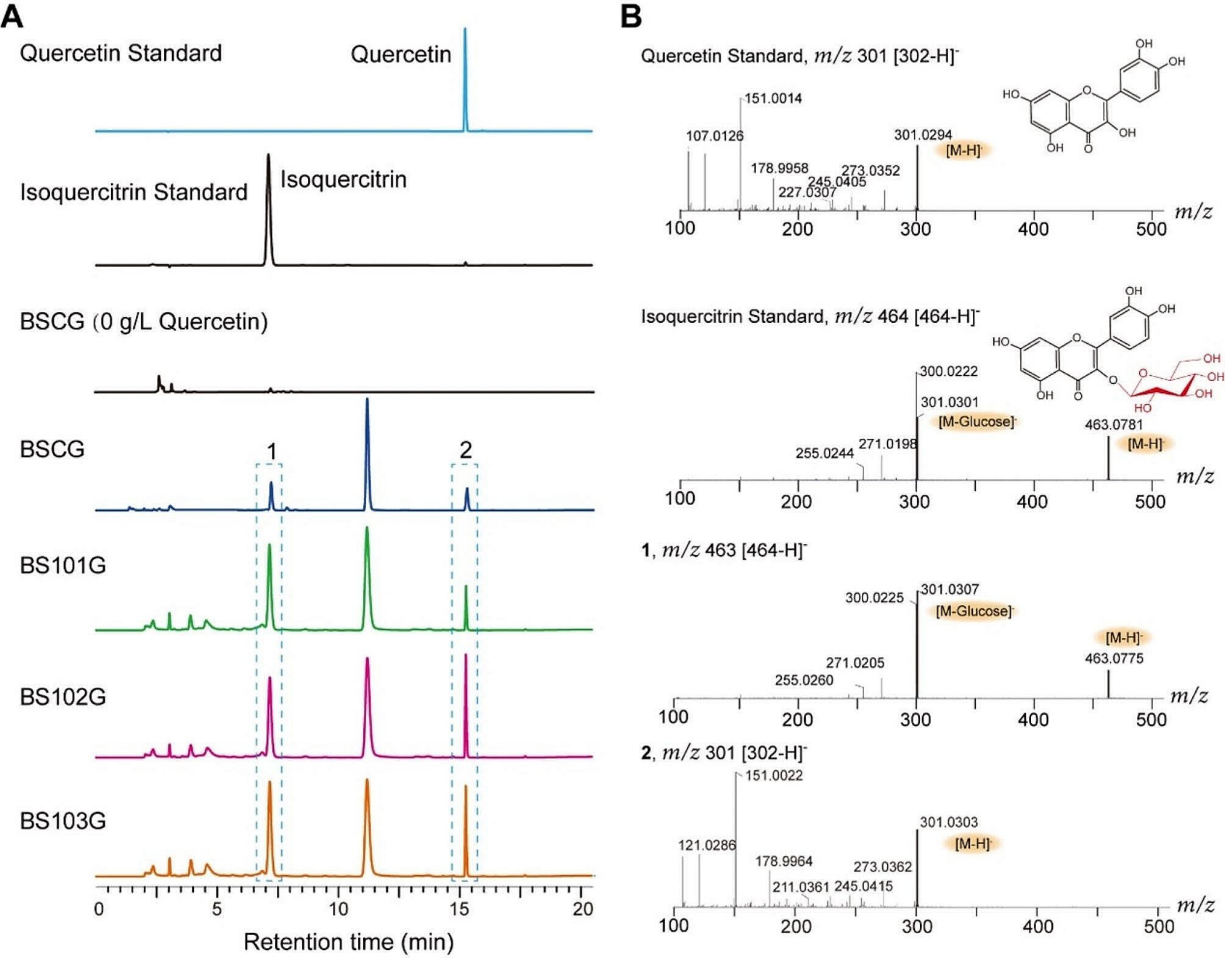
HPLC and LC-MS analysis of metabolites produced by the host *B. subtilis*. (**A**) HPLC spectra of quercetin, isoquercitrin and their standards. (**B**) LC-MS analysis results of quercetin, isoquercitrin in negative ion mode

According to HPLC analysis, three new peaks appeared in the UGT78D2 overexpressing strain BSCG following the addition of quercetin (Fig. 2A). A peak with a mass-to-charge ratio (*m/z*) value ([M-H]^−^=301.02), and another one with *m/z* value ([M-H]^−^=463.07) were detected in the strain BSCG, respectively (Fig. 2B). Those two peaks were identified as quercetin and isoquercitrin, respectively, when compared with the standards (Fig. 2B). Previous studies have indicated that the glycosyltransferase UGT78D2 exhibits an approximately 100-fold higher affinity for UDP-Glc than UDP-acetylglucosamine [14]. There was no quercetin-3-acetylglucosamine signal detected in UGT78D2 overexpressing strains. However, an unknown product was observed in these UGT78D2 overexpressing strains, which did not match the peak time of isoquercitrin standard (Fig. 2A). The characteristic fragment of [M-H]^−^ for this peak identified by LC-MS had an *m/z* value of 305 (Fig. S2). It was speculated to be the degradation of quercetin which was reported before [29]. These results demonstrated that the degradation of quercetin limits the accumulation of isoquercitrin in *B. subtilis* recombinants.

### Identification and elimination of the degradation of quercetin

**Fig. 3:**
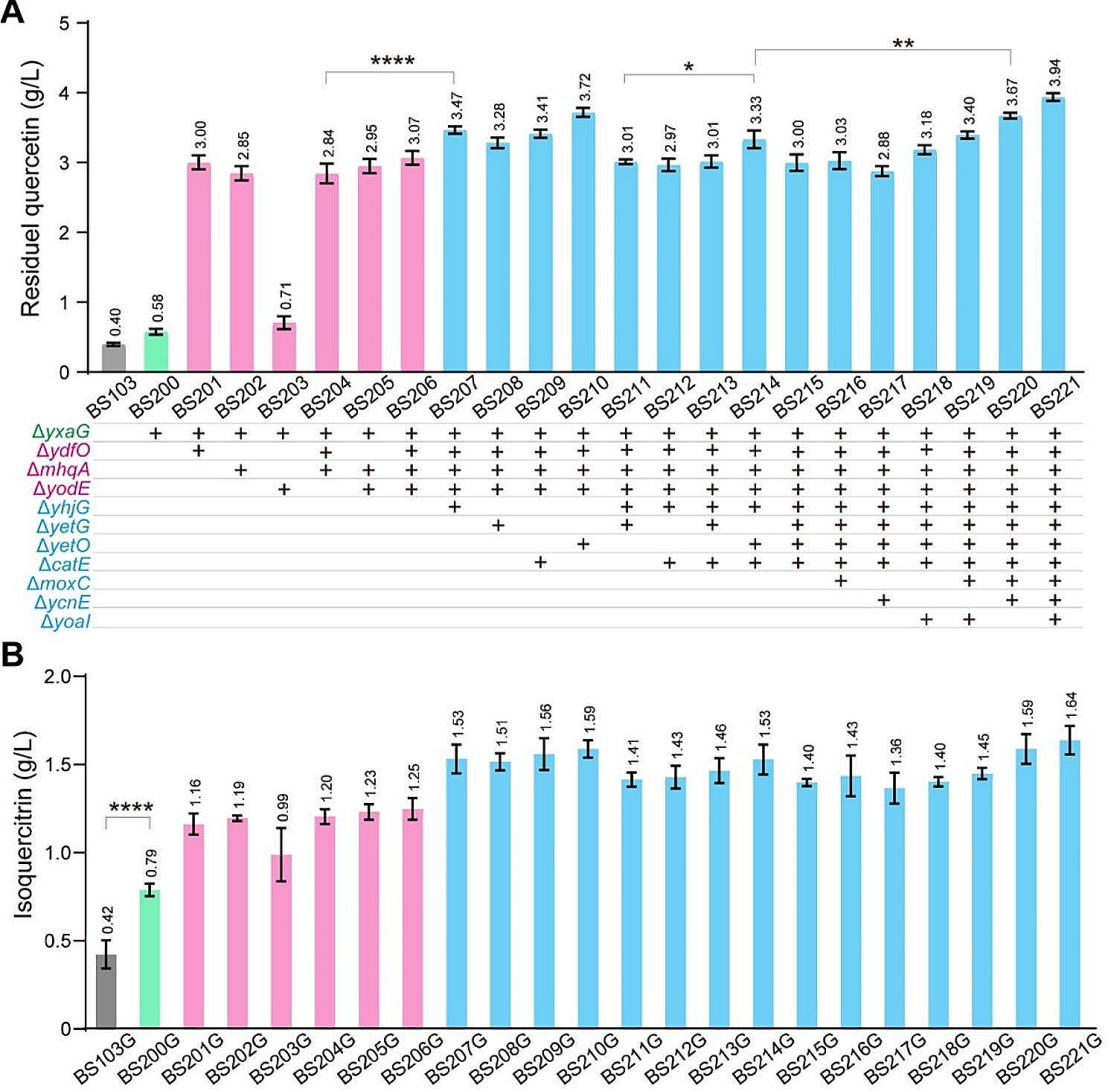
Influence of the proposed quercetin degradation pathway genes disruption on isoquercitrin accumulation. (**A**) Changes of residual quercetin titer by deleting proposed quercetin degradation pathway genes. *yxaG*, encoding quercetin 2,3-dioxygenase; *ydfO*, encoding ring-cleaving dioxygenase MhqO; *mhqA*, encoding ring-cleaving dioxygenase MhqA; *yodE*, encoding ring-cleaving dioxygenase MhqE; *yhjG*, encoding aromatic compound monooxygenase; *yetG*, encoding monooxygenase; *yetO*, encoding bifunctional P-450/NADPH-P450 reductase; *catE*, encoding catechol-2,3-dioxygenase; *moxC*, encoding monooxygenase; *ycnE*, encoding monooxygenase; *yoaI*, encoding 4-hydroxyphenylacetate 3-monooxygenase; (**B**) Changes of isoquercitrin titer by deleting proposed quercetin degradation pathway genes in UGT78D2 overexpressing strains

In strain BS103G, the isoquercitrin titer was only 0.42 g/L with a residual quercetin titer of 0.4 g/L, indicating that up to 80% of quercetin was degraded during incubation. These results suggested that the degradation of quercetin is the rate-limiting step in isoquercitrin biosynthesis. To overcome this issue, we investigated the enzymes associated with the degradation of quercetin, including ring-cleavage dioxygenases, and nonspecific oxidoreductases. Several candidate genes were selected to investigate the degradation of quercetin with the addition of 4 g/L quercetin in shake flask. Based on previous reports, it has been identified that the *yxaG* gene encodes quercetin 2,3-dioxygenase in *B. subtilis*, which is responsible for quercetin degradation [29, 30]. Firstly, the *yxaG* gene was deleted in BS200, resulting in a 45.0% increase in residual quercetin titer compared with BS103 (Fig. 3A). Secondly, three nonspecific ring-cleavage dioxygenase genes (*ydfO*, *mhqA*, *yodE* encoding ring-cleaving dioxygenase MhqO, MhqA, MhqE, respectively) were separately deleted in BS200, and the resulting strains were named as BS201 (Δ*ydfO*), BS202 (Δ*mhqA*), BS203 (Δ*yodE*), BS204 (Δ*ydfO*Δ*mhqA*), BS205 (Δ*mhqA*Δ*yodE*), and BS206 (Δ*ydfO*Δ*mhqA*Δ*yodE*). Compared with BS200, the residual quercetin titer increased by 417.2% in BS201 (3.00 g/L), 391.3% in BS202 (2.85 g/L), 22.4% in BS203 (0.71 g/L), 389.6% in BS204 (2.84 g/L), 408.6% in BS205 (2.95 g/L), and 429.3% in BS206 (3.07 g/L) (Fig. 3A). These results demonstrated that the degradation of quercetin was dramatically decreased but not completely eliminated in ring-cleavage dioxygenases triple mutant BS206.

Previous studies have demonstrated that these ring-cleavage dioxygenase genes were generally accompanied by oxidoreductase genes in same operon involved in degradation of aromatic compounds in bacteria [31, 32]. To investigate whether deletion of the oxidoreductases could reduce the degradation of quercetin, we chose seven oxidoreductases as candidates. Four oxidoreductase genes (*yhjG*, encoding aromatic compound monooxygenase; *yetG*, encoding monooxygenase; *yetO*, encoding bifunctional P450/NADPH-P450 reductase; *catE*, encoding catechol-2,3-dioxygenase) were individually deleted in BS206, generating strains BS207 (Δ*yhjG*), BS208 (Δ*yetG*), BS209 (Δ*yetO*), BS210 (Δ*catE*) (Fig. 3A). Compared with BS206, the residual quercetin titers increased by 13.0% in BS207, 6.8% in BS208, 11.1% in BS209, 21.2% in BS210 (Fig. 3A). However, no significant changes were observed regarding residual quercetin titers when multiple deletions were introduced into strains, such as BS211 (Δ*yhjG*Δ*yetG*), BS212 (Δ*yhjG*Δ*catE*), BS213 (Δ*yhjG*Δ*yetG*Δ*catE*), BS214 (Δ*yhjG*Δ*yetO*Δ*catE*), and BS215 (Δ*yhjG*Δ*yetG*Δ*yetO*Δ*catE*) (Fig. 3A). Subsequently, three additional oxidoreductase genes (*moxC*, encoding monooxygenase; *ycnE*, encoding monooxygenase; *yoaI*, encoding 4-hydroxyphenylacetate 3-monooxygenase) were successively deleted from BS215, generating strains BS216 (Δ*moxC*), BS217 (Δ*ycnE*), BS218 (Δ*yoaI*), BS219 (Δ*moxC*Δ*yoaI*), BS220 (Δ*moxC*Δ*ycnE*), and BS221 (Δ*moxC*Δ*ycnE*Δ*yoaI*). The residual quercetin titers were 3.03 g/L in BS216, 2.88 g/L in BS217, 3.18 g/L in BS218, 3.40 g/L in BS219, 3.67 g/L in BS220, and 3.94 g/L in BS221 (Fig. 3A). These results clearly demonstrated the effective inhibition of quercetin degradation observed specifically in strain BS221 (Fig. S3).

Next, to test whether the isoquercitrin titers change after releasing the rate-limiting step by reducing the degradation of quercetin, the p*P*_*43*_-UGT78D2 was transformed into these mutants. In BS200G, the isoquercitrin titer increased to 1.88-fold from 0.42 g/L (BS103G) to 0.79 g/L (Fig. 3B). The ring-cleavage dioxygenase genes were further deleted to reduce the ring-cleavage of quercetin. As a result, the isoquercitrin titers increased to 1.16 g/L in BS201G, 1.19 g/L in BS202G, 0.99 g/L in BS203G, 1.20 g/L in BS204G, 1.23 g/L in BS205G, and 1.25 g/L in BS206G (Fig. 3B). The influence of oxidoreductase disruptions on the synthesis of isoquercitrin was subsequently investigated. In the oxidoreductase disrupted strains, the isoquercitrin titers increased to 1.53 g/L in BS207G, 1.51 g/L in BS208G, 1.56 g/L in BS209G, 1.59 g/L in BS210G, 1.41 g/L in BS211G, 1.43 g/L in BS212G, 1.46 g/L in BS213G, 1.53 g/L in BS214G, 1.40 g/L in BS215G, 1.43 g/L in BS216G, 1.36 g/L in BS217G, 1.40 g/L in BS218G, 1.45 g/L in BS219G, 1.59 g/L in BS220G, and 1.64 g/L in BS221G (Fig. 3B). Moreover, biomass was slightly decreased by multiple oxidoreductase disruptions in BS221G compared with that of BS206G (Fig. S4). Overall, these results indicated that the degradation of quercetin is rate-limiting step and isoquercitrin titers can be further improved by blocking the degradation of quercetin.

### Reducing the deglycosylation of isoquercitrin and improving the glycosylation of quercetin

**Fig. 4:**
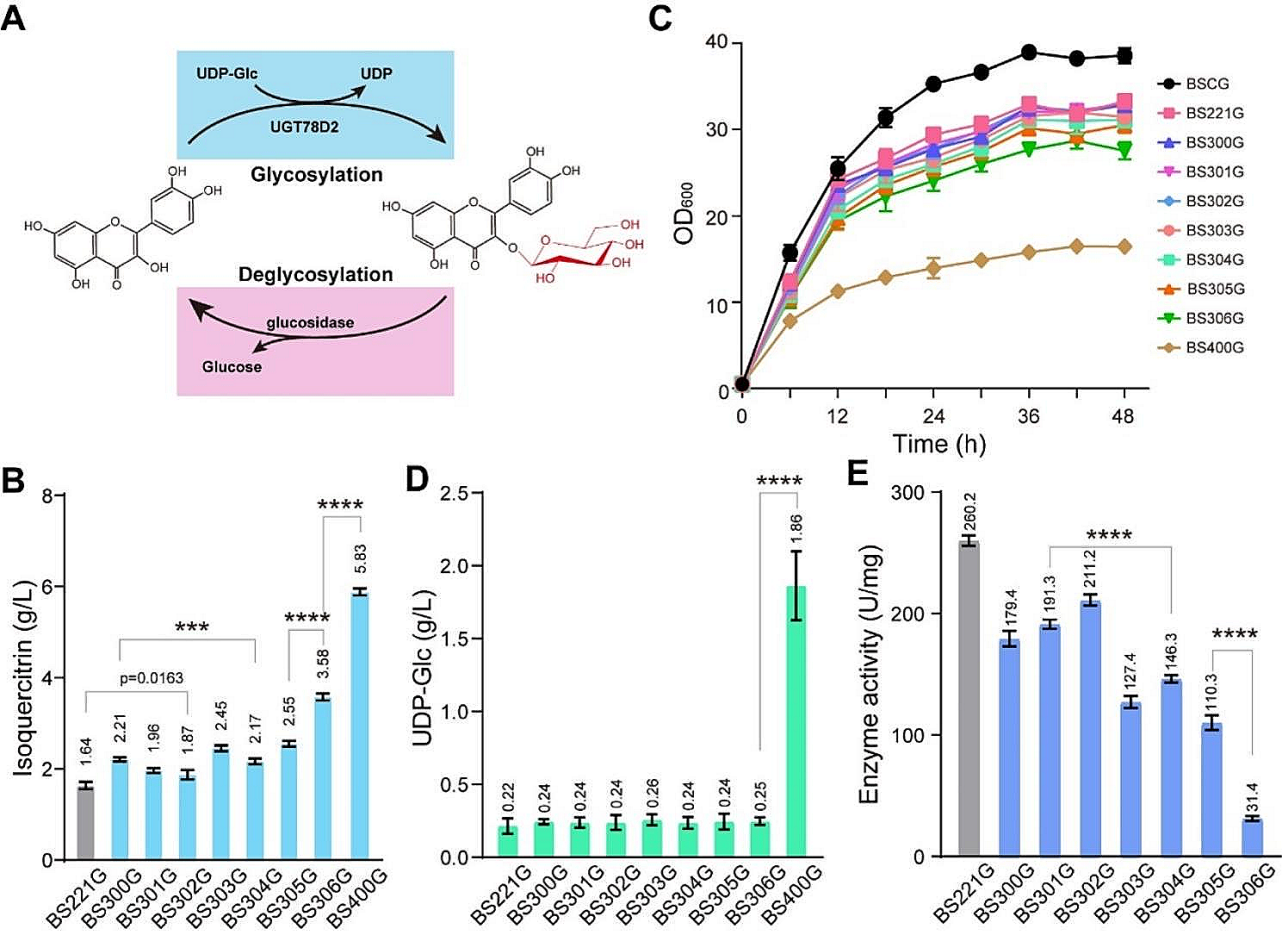
Reducing the deglycosylation of isoquercitrin and enhancing the glycosylation of quercetin to improve glycosides accumulation. (**A**) The deglycosylation of isoquercitrin and the glycosylation of quercetin; (**B**) The biomass of glucosidases deletion in *B. subtilis* recombinants BSCG (initial host), BS221G (control), BS300G (Δ*bglA*), BS301G (Δ*bglH*), BS302G (Δ*bglC*), BS303G (Δ*bglA*Δ*bglC*), BS304G (Δ*bglC*Δ*bglH*), BS305G (Δ*bglA*Δ*bglH*), BS306G (Δ*bglA*Δ*bglC*Δ*bglH*); (**C**) Changes of isoquercitrin titer by deleting glucosidases; (**D**) The concentrations of intracellular UDP-Glc in glucosidase disruption strains; (**E**) Changes of glucosidases activity by glucosidases disruption

In strain BS221G, the isoquercitrin titer was only 1.64 g/L, with the mole ratio of isoquercitrin to its precursor quercetin was approximately 1:2.7, indicating that the synthesis of isoquercitrin from quercetin is a key rate-limiting step (Fig. 3B). Further investigation is required to determine whether the insufficient UDP-Glc supply in the forward reaction or deglycosylation of isoquercitrin in the reverse reaction hinders the synthesis progress (Fig. 4A). Previous studies have reported three β-glucosidase genes (*bglH*, *bglA*, *bglC*) from *B. subtilis* with high activity in hydrolyzing isoflavone glucosides [33–35]. Therefore, these three β-glucosidase genes were individually deleted in BS221 to investigate their influence on the synthesis of isoquercitrin from quercetin. Compared with BS221G, the isoquercitrin titer increased by 34.7% to 2.21 g/L in BS300G strain (Δ*bglA*), 19.5% to 1.96 g/L in BS301G strain (Δ*bglH*), 14.0% to 1.87 g/L in BS302G strain (Δ*bglC*), 49.4% to 2.45 g/L in BS303G strain (Δ*bglA*Δ*bglC*), 32.3% to 2.17 g/L in BS304G strain (Δ*bglC*Δ*bglH*), 55.5% to 2.55 g/L in BS305G strain (Δ*bglA*Δ*bglH*), 118.3% to 3.58 g/L in BS306G strain (Δ*bglA*Δ*bglC*Δ*bglH*) (Fig. 4B). Compared to the control strain BS221G, these engineered strains exhibited a similar cell growth curve, indicating a negligible cell growth burden (Fig. 4C). And there was no significant change in the concentration of UDP-Glc in vivo among these strains (Fig. 4D). In addition, intracellular β-glucosidase activity was analyzed using the isoquercitrin as substrate. Compared with BS221G, the β-glucosidase activities were decreased by 31.1% to 179.4 U/mg in BS300G strain (Δ*bglA*), 26.5% to 191.3 U/mg in BS301G strain (Δ*bglH*), 18.8% to 211.2 U/mg in BS302G strain (Δ*bglC*), 51.0% to 127.4 U/mg in BS303G strain (Δ*bglA*Δ*bglC*), 43.8% to 146.3 U/mg in BS304G strain (Δ*bglC*Δ*bglH*), 57.6% to 110.3 U/mg in BS305G strain (Δ*bglA*Δ*bglH*), and 87.9% to 31.4 U/mg in BS306G strain (Δ*bglA*Δ*bglC*Δ*bglH*) (Fig. 4E). Overall, these results indicate that β-glucosidase is the major enzyme for the deglycosylation of isoquercitrin in *B. subtilis*.

Next, to investigate whether sufficient UDP-Glc supply could improve glycosylation of quercetin for isoquercitrin synthesis, the carbon flux was redistributed towards UDP-Glc synthesis by deleting the glucose-6-phosphate isomerase gene (*pgi*) in BS306G, generating the strain BS400G. Because PGI mediates the carbon flux distribution among pentose phosphate pathway, glycolysis pathway, and UDP-Glc synthesis pathway through catalyzing the interconversion of glucose-6-phophate (the starting metabolite of pentose phosphate pathway and UDP-Glc synthesis pathway) and fructose-6-phosphate (the starting metabolite of glycolysis pathway) [28, 36, 37]. Compared with BS306G, the isoquercitrin titer increased by 62.8% to 5.83 g/L with a yield on quercetin of 97% (Fig. 4B). As a result, cell growth was significantly decreased by *pgi* disruption, and the concentration of intracellular UDP-Glc increased by 7.44-fold to 1.86 g/L in BS400G (Fig. 4CD). Therefore, our results further prove that the insufficient UDP-Glc supply is a rate-limiting step for isoquercitrin production.

**Fig. 5:**
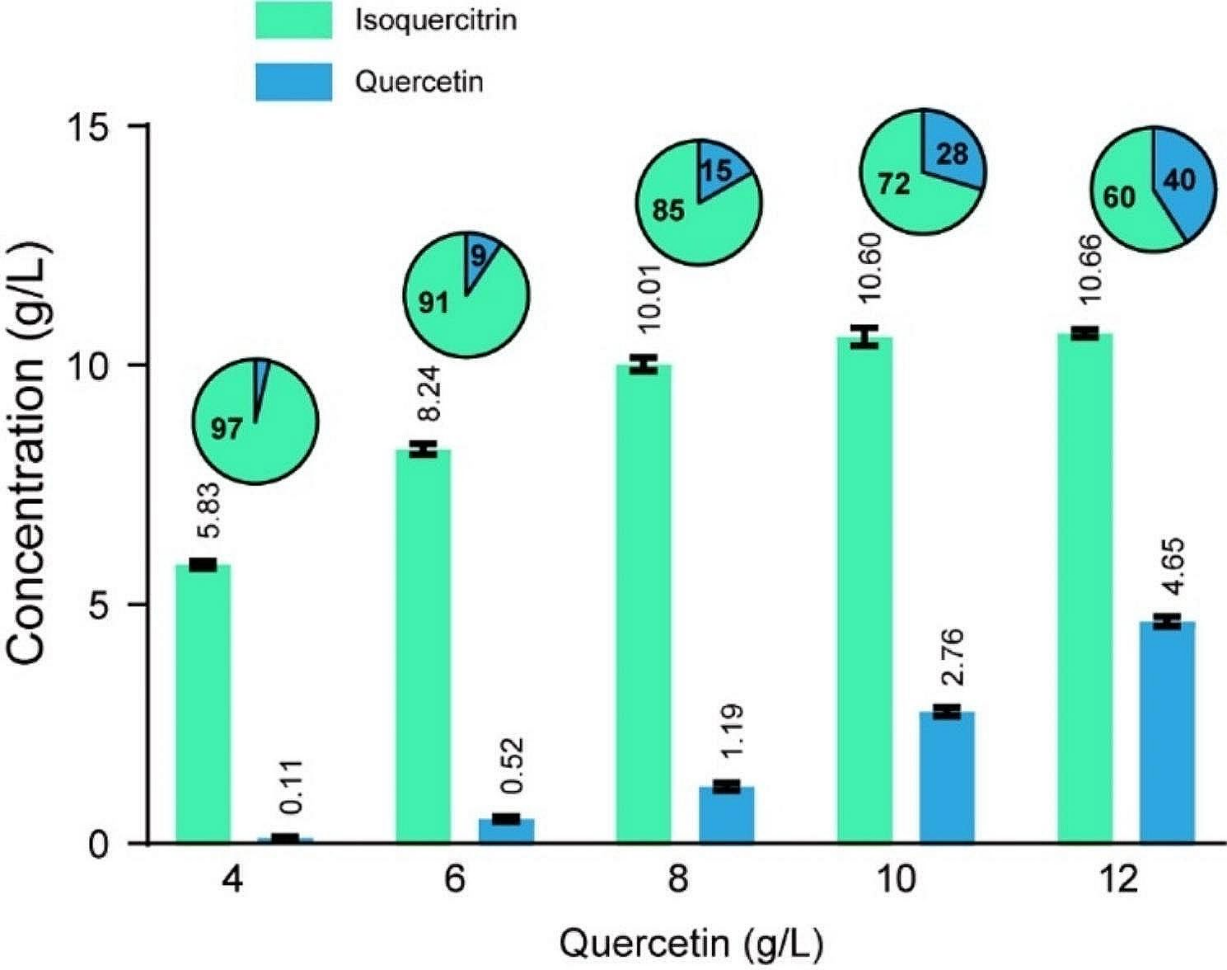
Effect of the addition of quercetin on the synthesis of isoquercitrin by BS400G for 48 h. Pie charts show the proportions of isoquercitrin and quercetin molar concentration

According to the above results, the conversion of quercetin was up to 97% after 48 h of incubation when 4 g/L quercetin was supplemented. To explore the potential of *B. subtilis* recombinants to produce isoquercitrin, the addition of quercetin in the BS400G culture was examined (Fig. 5). After incubation of 48 h, the isoquercitrin titer increased to 8.24 g/L (yield 91%), 10.01 g/L (yield 85%), 10.60 g/L (yield 72%), and 10.66 g/L (yield 60%), with the 6 g/L, 8 g/L, 10 g/L, and 12 g/L quercetin supplemented, respectively (Fig. 5).

### Isoquercitrin production via fed-batch fermentation

**Fig. 6:**
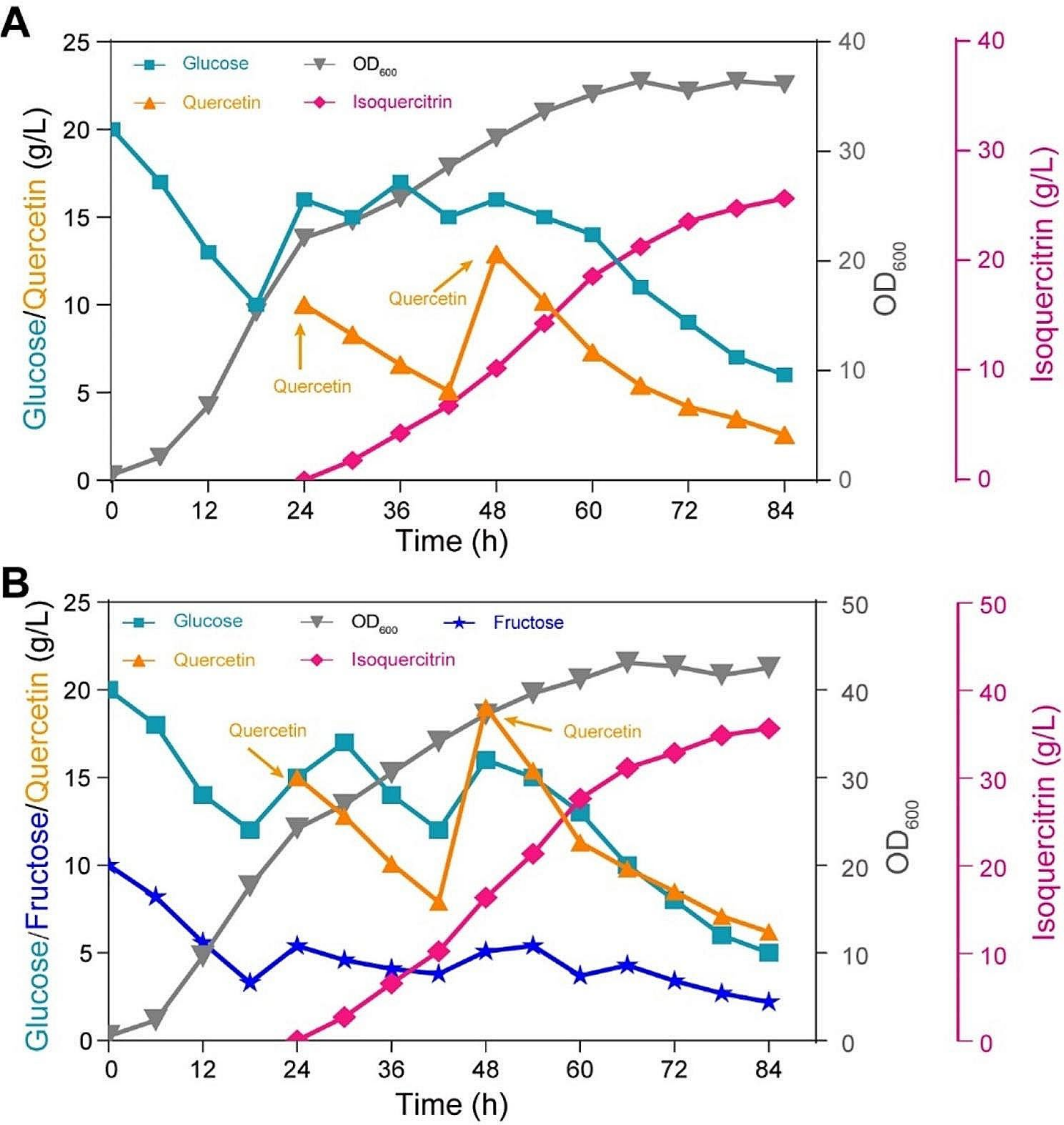
Fed-batch fermentation for isoquercitrin production by strain BS400G in a 1.3-L fermentor. Growth profiles and glucose, quercetin consumption curves during fed-batch fermentation. (**A**) Feeding glucose at adjusted feeding rate for cell growth in fermentor. (**B**) Feeding glucose and fructose at adjusted feeding rate for cell growth in fermentor

To further evaluate the fermentation characteristics of the engineered strain BS400G for biosynthesis of isoquercitrin from quercetin, an 84-h fermentation progress in a 1.3-L fermentor was performed. As shown in Fig. 6A, cell growth showed a rapid increase and glucose was rapidly consumed during exponential-phase. Glucose concentrations were kept below 20 g/L through adjusted feeding rate. The maximal OD_600_ of strain BS400G reached 36.4 at 66 h. However, the biomass entered a stage of slow increase after 24 h following the feeding of quercetin, the results demonstrated that the feeding quercetin was toxic to cell growth. Therefore, to further enhance the isoquercitrin titer, quercetin can be supplied using an appropriate feeding approach. The maximal isoquercitrin titer was 25.7 g/L with the yield on quercetin of 83.6% at 84 h.

To release the inhibition of cell growth by *pgi* disruption, the biomass was further increased by 18.4% with feeding glucose and fructose at adjusted feeding rates (Fig. 6B). As a result, the maximal OD_600_ of strain BS400G reached 43.1 and the maximal isoquercitrin titer achieved 35.6 g/L with the yield on quercetin of 77.2%. Isoquercitrin productivity was enhanced to 0.59 g/L/h in fed-batch culture, which was 2.1-fold that in batch fermentation (0.28 g/L/h).

## Discussion

When *B. subtilis* is exposed to various toxic compounds in the soil, making it have different specific detoxification systems. The *mhqED*, *ydfNOP* operons, and *mhqA* have been characterized to encode hydroquinone-specific extradiol cleavage dioxygenases [31]. And our findings demonstrated that the degradation of quercetin can be reduced by disrupting three ring-cleavage dioxygenases (Fig. 3A). These three dioxygenases might be involved in the ring-cleavage of quercetin. However, the disruption of three ring-cleavage dioxygenases could not completely block the degradation of quercetin. Ring-cleavage dioxygenases play a role of ring-cleavage after the hydroxylation of aromatic compounds [38]. Therefore, oxidoreductases were selected as potential candidates to investigate the hydroxylation of aromatic compounds. In our work, multiple oxidoreductases disrupting strains (BS211, BS212, BS213) consumed more quercetin compared to only one oxidoreductase deleted strains (BS207, BS208, BS209, BS210) (Fig. 3A). Several metabolic operons in *B. subtilis*, such as *yfiDE*, *yfdNOP*, *mhqED*, *ycnDE*, *yodC*, and *mhqA*, were found to be differentially activated by 2-methylhydroquinone, catechol, and chromanon [31]. This could be attributed to the modulation of expression levels of multiple detoxification systems in response to various aromatic compounds in *B. subtilis*. Further work is required to characterize the functions of the oxidoreductases in the process of quercetin degradation. In conclusion, although the mechanism underlying quercetin catabolism remains unclear, our work proved that it is feasible to produce high amounts of isoquercitrin in *B. subtilis*.

*B. subtilis* harbors many intracellular glucosidases for the hydrolysis of glycosidic bonds. Two aryl-phospho-β-D-glucosidases (BglH and BglC) have been identified in *B. subtilis*, capable of cleaving the β-glycosidic bond of isoflavone glycoside [33]. Interestingly, BglA and BglH are the major β-glucosidases responsible for MUG-P (4-Methylumbelliferyl-β-D-glucopyranoside-6-phosphate) hydrolysis by extracts derived from *B. subtilis* cells in late-exponential-phase and stationary-phase [34]. BglH was synthesized in all growth stages, and BglA synthesis increased in exponential-phase, while BglC highest in stationary-phase [34]. And then, gene inactivation studies revealed that the major β-glucosidase encoded by *bglC* in *B. subtilis* 168 contributed about half of the total glucosidase activity and that other β-glucosidase genes *bglA* and *bglH*, all barely committed to hydrolyze isoflavone glycosides [35]. However, the regulatory mechanism controlling the synthesis of BglC, BglH, BglA as glucosidases at different stages of growth remain unclear. Moreover, it has been verified that the synthesis of BglH and BglA can be induced by various glycosides [34]. Notably, the expression of *bglC* alone allows cells to grow on arbutin or salicin as the sole carbon source [34]. Our results highlight that the strain BS306G with triple deletions of *bglC*, *bglH*, and *bglA* exhibits a significant decrease in the hydrolysis of isoquercitrin and while promotes product accumulation. These results present an efficient strategy for overcoming the deglycosylation exerted by unspecific β-glucosidase in glycoside biosynthesis.

Many strategies have been employed to increase the supply of UDP-sugar, such as remodeling UDP-sugar biosynthesis pathways, decreasing consumption of UDP-sugar in non-essential pathway, and strengthening regeneration of UDP-sugar by increasing UDP recovery and utilization [39–41]. Insufficient supply of UDP-Glc has been identified as a rate-limiting step in biosynthesis of glycosides. Here, we enhanced intracellular UDP-Glc pool and increased isoquercitrin production by combination of reconstructing the UDP-Glc synthesis pathway and redistributing carbon flow towards UDP-Glc synthesis through *pgi* disruption. These results proved the high-effective system of isoquercitrin biosynthesis in *B. subtilis*. Further studies can be performed to balance carbon flow between cell growth and UDP-Glc synthesis through optimizing the expression of *pgi*. In particular, for the *pgi* disrupting strain BS400G, and while the UDP-Glc titer was increased to 7.44-fold, cell growth was severely inhibited. Therefore, future experiments should be focused on how to control the expression of *pgi*, and to improve the UDP-Glc supply using dynamic regulation and strengthening UDP recovery and utilization.

## Conclusion

In this study, a *B. subtilis* cell factory for isoquercitrin production from quercetin was constructed for the first time. Enhancing the supply of UDP-Glc alongside eliminating the degradation of quercetin and reducing the hydrolysis of isoquercitrin increased the isoquercitrin titer by ∼ 29 times to 10.6 g/L. Subsequently, glucose and fructose feeding strategy increased the isoquercitrin titer to 35.6 g/L in a 1.3-L fermentor. The strategies proposed in this study provides a reference to improve the production of other flavonoid glycosides by engineered *B. subtilis* cell factories.

### Electronic supplementary material

Below is the link to the electronic supplementary material.

Supplementary Material 1

## Data Availability

No datasets were generated or analysed during the current study.

## Abbreviations

UDP-Glc: UDP-glucose
*pgi*: encoding glucose-6-phosphate isomerase
Isoquercitrin: quercetin-3-*O*-β-d-glucopyranoside
GRAS: generally recognized as safe
*yxaG*: quercetin dioxygenase gene
PgcA: glucose phosphate mutase
GtaB: gucose-1-phosphate uridyltransferase
*ydfO*: encoding ring-cleaving dioxygenase MhqO
*mhqA*: encoding ring-cleaving dioxygenase MhqA
*yodE*: encoding ring-cleaving dioxygenase MhqE
*yhjG*: encoding aromatic compound monooxygenase
*yetG*: encoding monooxygenase
*yetO*: encoding bifunctional P450/NADPH-P450 reductase
*catE*: encoding catechol-2,3-dioxygenase
*moxC*: encoding monooxygenase
*ycnE*: encoding monooxygenase
*yoaI*: encoding 4-hydroxyphenylacetate 3-monooxygenase
*bglA*: aryl-phospho-β-D-glucosidase BglA
*bglH*: aryl-phospho-β-D-glucosidase BglH
*bglC*: aryl-phospho-β-D-glucosidase BglC
