# Supplementary material for: Combinatorial metabolic engineering of Bacillus subtilis enables the efficient biosynthesis of isoquercitrin from quercetin
Source: Microb Cell Fact. 2024 Apr 20;23:114. doi: 10.1186/s12934-024-02390-5 (PMC11031953; doi:10.1186/s12934-024-02390-5)
Supplement: Supplementary file 1 — Supplementary Material 1 [file 12934_2024_2390_MOESM1_ESM.docx]

**Supplementary Information**

**Combinatorial metabolic engineering of *Bacillus subtilis* enables the efficient biosynthesis of isoquercitrin from quercetin**

Tengfei Niu^1,2^, Chaokang Huang^1^, Rufeng Wang^1,2,3^*, Li Yang^1,2,3^, Shujuan Zhao^1,2,3^, Zhengtao Wang^1,2,3^*

^1^ Institute of Chinese Materia Medica, Shanghai University of Traditional Chinese Medicine, Shanghai 201203, China

^2^ The SATCM Key Laboratory for New Resources & Quality Evaluation of Chinese Medicine, Institute of Chinese Materia Medica, Shanghai University of Traditional Chinese Medicine, Shanghai 201203, China

^3^ The MOE Key Laboratory for Standardization of Chinese Medicines and Shanghai Key Laboratory of Compound Chinese Medicines, Shanghai University of Traditional Chinese Medicine, Shanghai 201203, China

* Corresponding author:

Rufeng Wang

No. 1200 Cailun Road, Pudong new area, Shanghai 201203, China

Tel: +86-21-5132-2495; Fax: +86-21-5132-0840

E-mails: wrffrw0801@shutcm.edu.cn

Zhengtao Wang

No. 1200 Cailun Road, Pudong new area, Shanghai 201203, China

Tel: +86-21-5132-2507; Fax: +86-21-5132-2505

E-mail: ztwang@shutcm.edu.cn


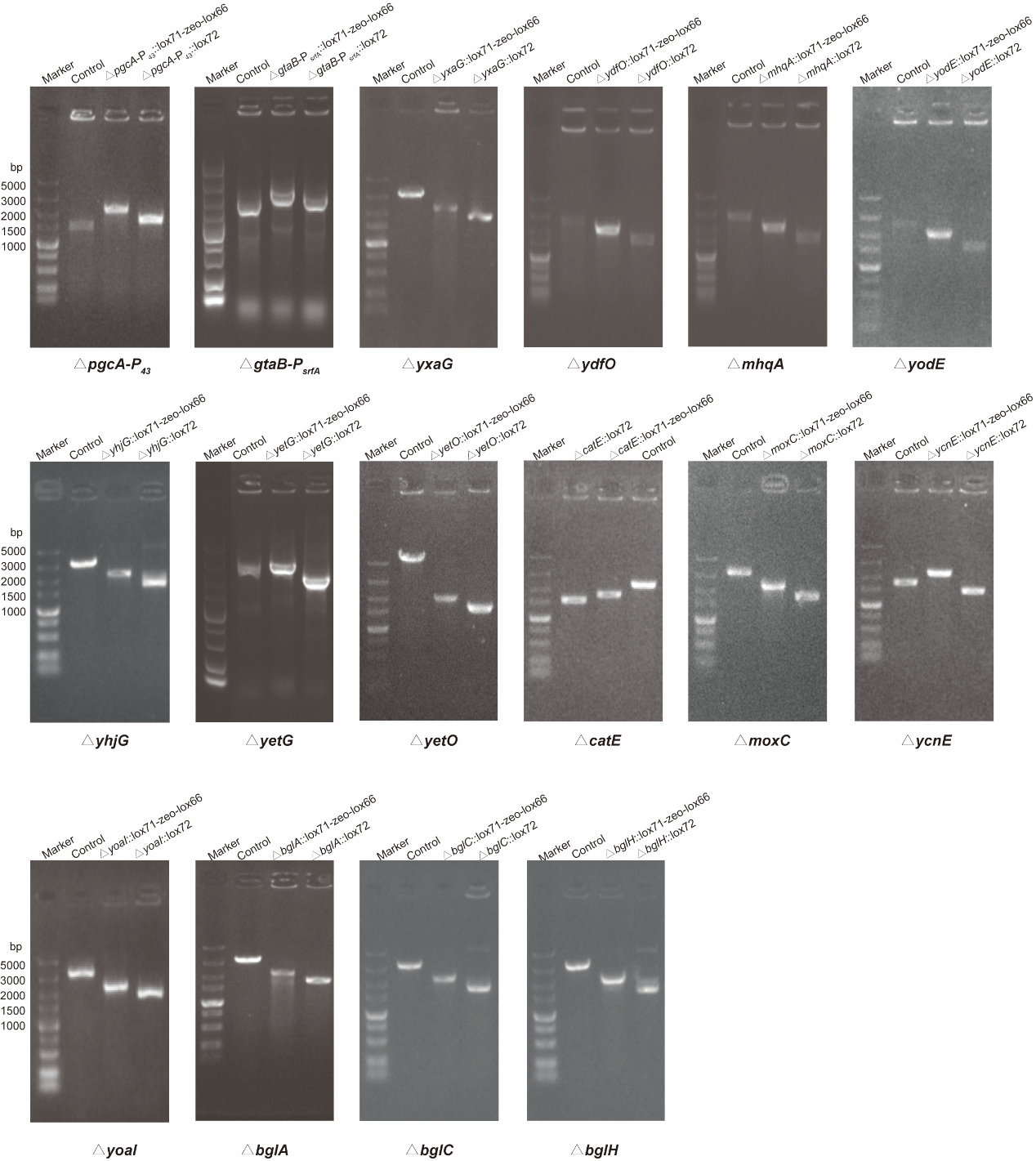


**Fig. S1** Confirmation of the gene knock-in and knock-out verified by PCR products.


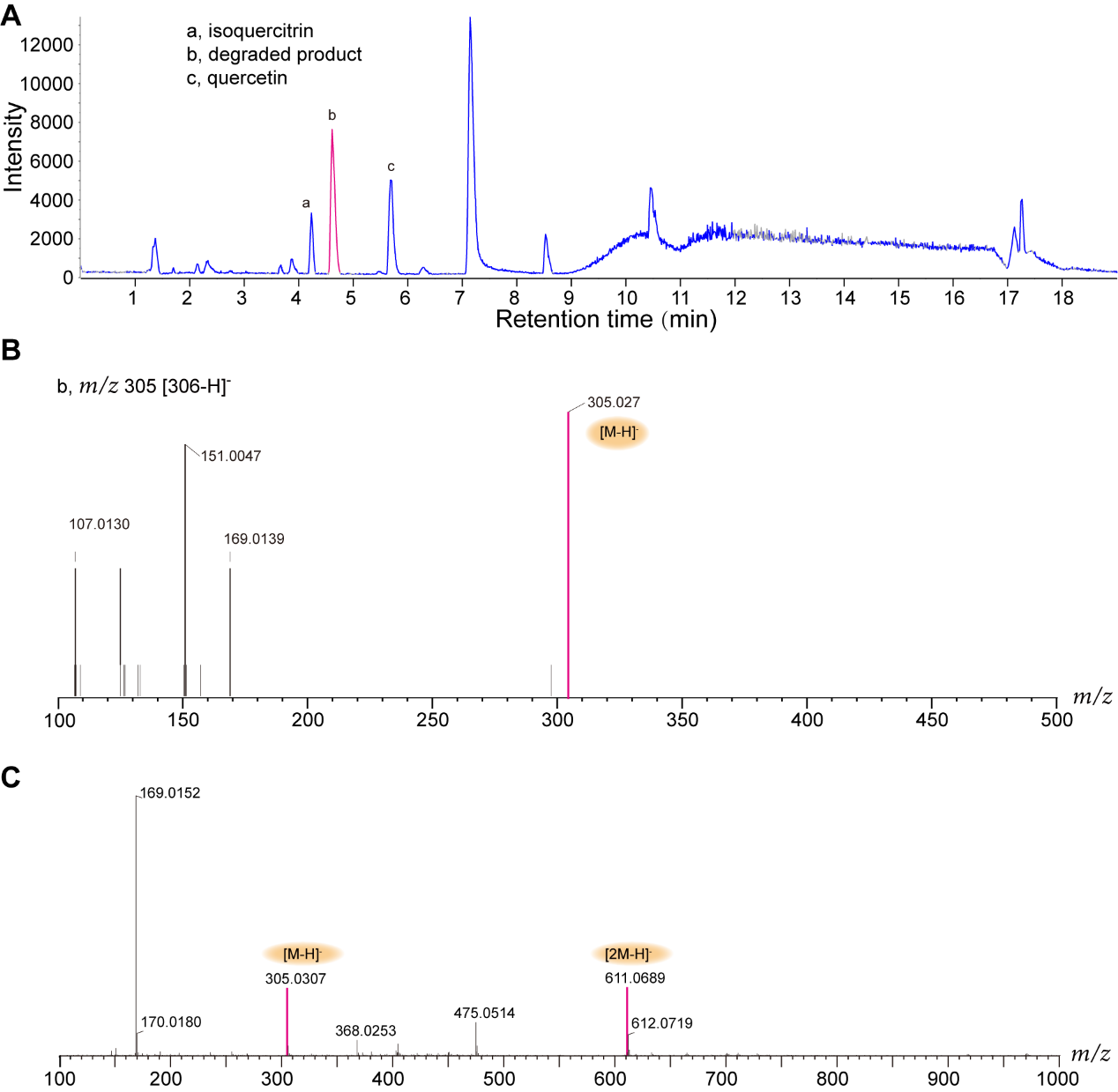


**Fig. S2** LC-MS analysis results of the degraded product in negative ion mode. (A) HPLC spectra of isoquercitrin (a), degrade product (b), and quercetin (c). (B) MS2 spectra of degraded product in negative ion mode. (C) MS1 spectra of degraded product in negative ion mode.


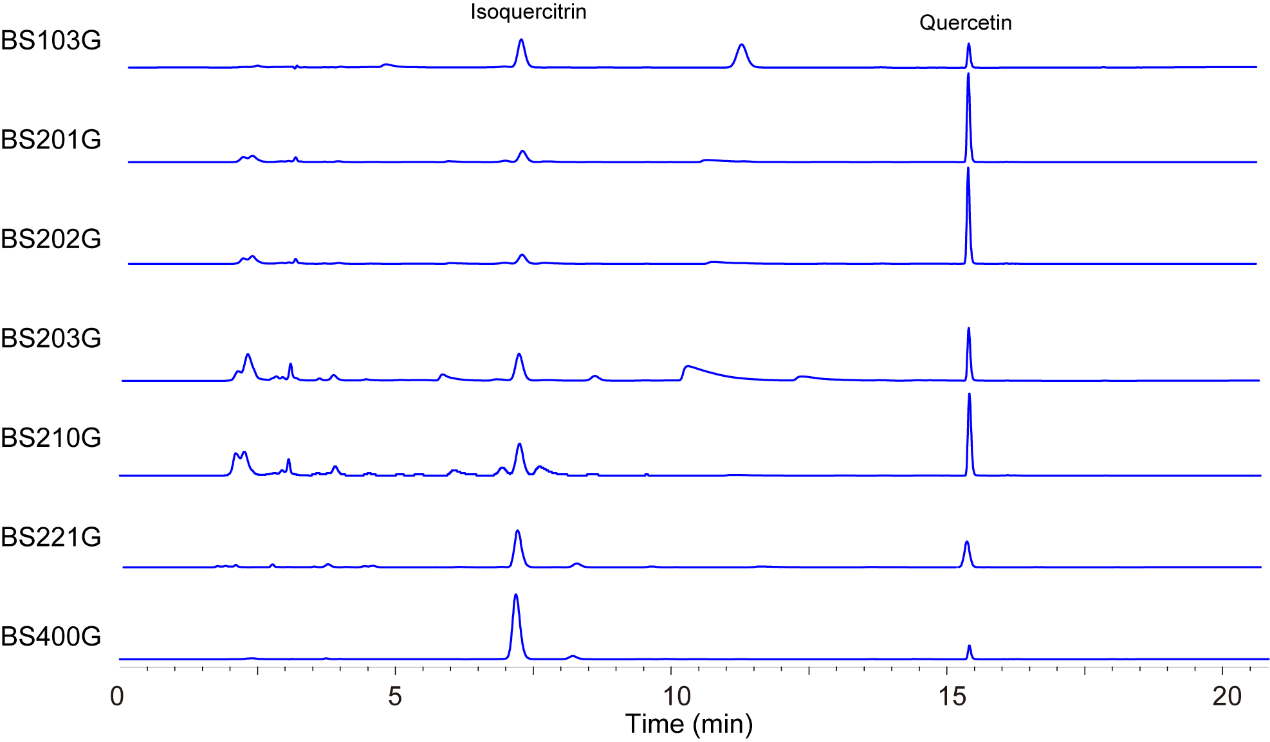


**Fig. S3** HPLC analysis of metabolites produced by the engineered *B. subtilis*.

**
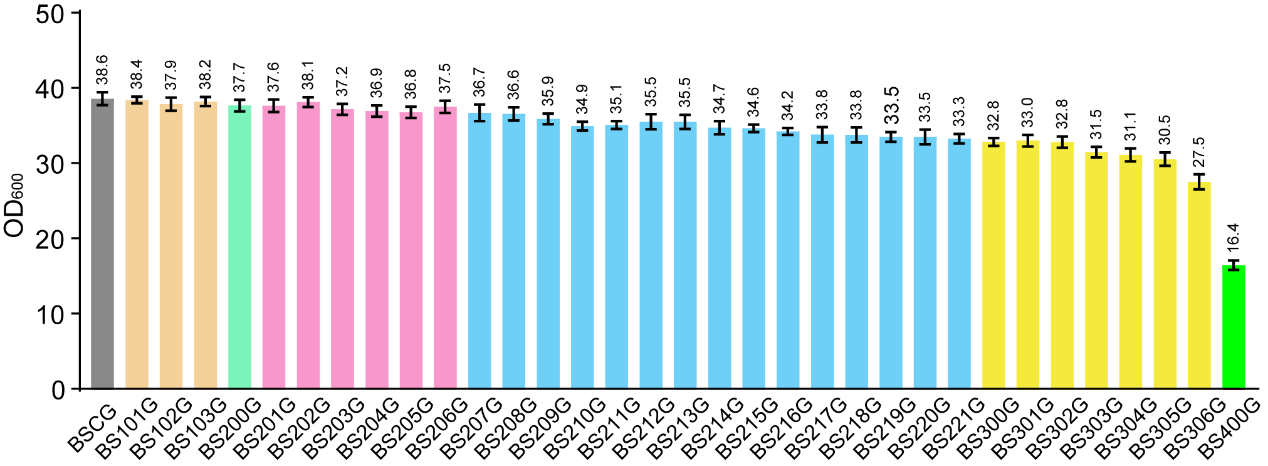
**

**Fig. S4** The biomass of engineered *B. subtilis* strains.

**Table S1** The sequence of codon-optimized gene UGT78D2.

| Gene name | Nucleotide sequence |
| --- | --- |
| UGT78D2 | ATGACCAAACCCTCCGACCCAACCAGAGACTCCCACGTGGCAGTTCTCGCTTTTCCTTTCGGCACTCATGCAGCTCCTCTCCTCACCGTCACGCGCCGCCTCGCCTCCGCCTCTCCTTCCACCGTCTTCTCTTTCTTCAACACCGCACAATCCAACTCTTCGTTATTTTCCTCCGGTGACGAAGCAGATCGTCCGGCGAACATCAGAGTATACGATATTGCCGACGGTGTTCCGGAGGGATACGTGTTTAGCGGGAGACCACAGGAGGCGATCGAGCTGTTTCTTCAAGCTGCGCCGGAGAATTTCCGGAGAGAAATCGCGAAGGCGGAGACGGAGGTTGGTACGGAAGTGAAATGTTTGATGACTGATGCGTTCTTCTGGTTCGCGGCTGATATGGCGACGGAGATAAATGCGTCGTGGATTGCGTTTTGGACCGCCGGAGCAAACTCACTCTCTGCTCATCTCTACACAGATCTCATCAGAGAAACCATCGGTGTCAAAGAAGTAGGTGAGCGTATGGAGGAGACAATAGGGGTTATCTCAGGAATGGAGAAGATCAGAGTCAAAGATACACCAGAAGGAGTTGTGTTTGGGAATTTAGACTCTGTTTTCTCAAAGATGCTTCATCAAATGGGTCTTGCTTTGCCTCGTGCCACTGCTGTTTTCATCAATTCTTTTGAAGATTTGGATCCTACATTGACGAATAACCTCAGATCGAGATTTAAACGATATCTGAACATCGGTCCTCTCGGGTTATTATCTTCTACATTGCAACAACTAGTGCAAGATCCTCACGGTTGTTTGGCTTGGATGGAGAAGAGATCTTCTGGTTCTGTGGCGTACATTAGCTTTGGTACGGTCATGACACCGCCTCCTGGAGAGCTTGCGGCGATAGCAGAAGGGTTGGAATCGAGTAAAGTGCCGTTTGTTTGGTCGCTTAAGGAGAAGAGCTTGGTTCAGTTACCAAAAGGGTTTTTGGATAGGACAAGAGAGCAAGGGATAGTGGTTCCATGGGCACCGCAAGTGGAACTGCTGAAACACGAAGCAACGGGTGTGTTTGTGACGCATTGTGGATGGAACTCGGTGTTGGAGAGTGTATCGGGTGGTGTACCGATGATTTGCAGGCCATTTTTTGGGGATCAGAGATTGAACGGAAGAGCGGTGGAGGTTGTGTGGGAGATTGGAATGACGATTATCAATGGAGTCTTCACGAAAGATGGGTTTGAGAAGTGTTTGGATAAAGTTTTAGTTCAAGATGATGGTAAGAAGATGAAATGTAATGCTAAGAAACTTAAAGAACTAGCTTACGAAGCTGTCTCTTCTAAAGGAAGGTCCTCTGAGAATTTCAGAGGATTGTTGGATGCAGTTGTAAACATTATTTGA |

**Table S2** Strains and plasmids used in this study.

| Names | Characteristics | Reference |
| --- | --- | --- |
| strains |  |  |
| BSC | *B. subtilis* 168, starting strain | Lab stock |
| BS101 | *B. subtilis* 168 derivate, Δ*P*_P43_-*pgcA::lox72*, overexpression of *pgcA* gene under the control of promoter *P*_P43_ | This work |
| BS102 | *B. subtilis* 168 derivate, Δ*P*_srfA_-*gtaB::lox72*, overexpression of *gtaB* gene under the control of promoter *P*_srfA_ | This work |
| BS103 | *B. subtilis* 168 derivate, Δ*P*_P43_-*pgcA*Δ*P*_srfA_-*gtaB::lox72*, overexpression of *pgcA* gene and *gtaB* gene under the control of promoter *P*_P43_ and promoter *P*_srfA_, respectively | This work |
| BS200 | BS103 derivate, Δ*yxaG::lox72* | This work |
| BS201 | BS200 derivate, Δ*ydfO::lox72* | This work |
| BS202 | BS200 derivate, Δ*mhqA::lox72* | This work |
| BS203 | BS200 derivate, Δ*yodE::lox72* | This work |
| BS204 | BS200 derivate, Δ*mhqA*Δ*ydfO::lox72* | This work |
| BS205 | BS200 derivate, Δ*mhqA*Δ*yodE::lox72* | This work |
| BS206 | BS200 derivate, Δ*mhqA*Δ*yodE*Δ*ydfO::lox72* | This work |
| BS207 | BS200 derivate, Δ*mhqA*Δ*yodE*Δ*ydfO*Δ*yhjG::lox72* | This work |
| BS208 | BS200 derivate, Δ*mhqA*Δ*yodE*Δ*ydfO*Δ*yetG::lox72* | This work |
| BS209 | BS200 derivate, Δ*mhqA*Δ*yodE*Δ*ydfO*Δ*catE::lox72* | This work |
| BS210 | BS200 derivate, Δ*mhqA*Δ*yodE*Δ*ydfO*Δ*yetO::lox72* | This work |
| BS211 | BS200 derivate, Δ*mhqA*Δ*yodE*Δ*ydfO*Δ*yhjG*Δ*yetG::lox72* | This work |
| BS212 | BS200 derivate, Δ*mhqA*Δ*yodE*Δ*ydfO*Δ*yhjG*Δ*catE::lox72* | This work |
| BS213 | BS200 derivate, Δ*mhqA*Δ*yodE*Δ*ydfO*Δ*yhjG*Δ*catE*Δ*yetG::lox72* | This work |
| BS214 | BS200 derivate, Δ*mhqA*Δ*yodE*Δ*ydfO*Δ*yhjG*Δ*catE*Δ*yetO::lox72* | This work |
| BS215 | BS200 derivate, Δ*mhqA*Δ*yodE*Δ*ydfO*Δ*yhjG*Δ*catE*Δ*yetO*Δ*yetG::lox72* | This work |
| BS216 | BS200 derivate, Δ*mhqA*Δ*yodE*Δ*ydfO*Δ*yhjG*Δ*catE*Δ*yetO*Δ*yetG*Δ*moxC::lox72* | This work |
| BS217 | BS200 derivate, Δ*mhqA*Δ*yodE*Δ*ydfO*Δ*yhjG*Δ*catE*Δ*yetO*Δ*yetG*Δ*ycnE::lox72* | This work |
| BS218 | BS200 derivate, Δ*mhqA*Δ*yodE*Δ*ydfO*Δ*yhjG*Δ*catE*Δ*yetO*Δ*yetG*Δ*yoaI::lox72* | This work |
| BS219 | BS200 derivate, Δ*mhqA*Δ*yodE*Δ*ydfO*Δ*yhjG*Δ*catE*Δ*yetO*Δ*yetG*Δ*moxC*Δ*yoaI::lox72* | This work |
| BS220 | BS200 derivate, Δ*mhqA*Δ*yodE*Δ*ydfO*Δ*yhjG*Δ*catE*Δ*yetO*Δ*yetG*Δ*moxC*Δ*ycnE::lox72* | This work |
| BS221 | BS200 derivate, Δ*mhqA*Δ*yodE*Δ*ydfO*Δ*yhjG*Δ*catE*Δ*yetO*Δ*yetG*Δ*moxC*Δ*ycnE*Δ*yoaI::lox72* | This work |
| BS300 | BS221 derivate, Δ*bglA::lox72* | This work |
| BS301 | BS221 derivate, Δ*bglH::lox72* | This work |
| BS302 | BS221 derivate, Δ*bglC::lox72* | This work |
| BS303 | BS221 derivate, Δ*bglAbglC::lox72* | This work |
| BS304 | BS221 derivate, Δ*bglCbglH::lox72* | This work |
| BS305 | BS221 derivate, Δ*bglAbglH::lox72* | This work |
| BS306 | BS221 derivate, Δ*bglAbglCbglH::lox72* | This work |
| BS400 | BS306 derivate, Δ*pgi::lox72* | This work |
| BSCG | *B. subtilis* 168 derivate, overexpression of *Arabidopsis thaliana* UGT78D2 gene | This work |
| BS101G | BS101 derivate, overexpression of *Arabidopsis thaliana* UGT78D2 gene | This work |
| BS102G | BS102 derivate, overexpression of *Arabidopsis thaliana* UGT78D2 gene | This work |
| BS103G | BS103 derivate, overexpression of *Arabidopsis thaliana* UGT78D2 gene | This work |
| BS200G | BS200 derivate, overexpression of *Arabidopsis thaliana* UGT78D2 gene | This work |
| BS201G | BS201 derivate, overexpression of *Arabidopsis thaliana* UGT78D2 gene | This work |
| BS202G | BS202 derivate, overexpression of *Arabidopsis thaliana* UGT78D2 gene | This work |
| BS203G | BS203 derivate, overexpression of *Arabidopsis thaliana* UGT78D2 gene | This work |
| BS204G | BS204 derivate, overexpression of *Arabidopsis thaliana* UGT78D2 gene | This work |
| BS205G | BS205 derivate, overexpression of *Arabidopsis thaliana* UGT78D2 gene | This work |
| BS206G | BS206 derivate, overexpression of *Arabidopsis thaliana* UGT78D2 gene | This work |
| BS207G | BS207 derivate, overexpression of *Arabidopsis thaliana* UGT78D2 gene | This work |
| BS208G | BS208 derivate, overexpression of *Arabidopsis thaliana* UGT78D2 gene | This work |
| BS209G | BS209 derivate, overexpression of *Arabidopsis thaliana* UGT78D2 gene | This work |
| BS210G | BS210 derivate, overexpression of *Arabidopsis thaliana* UGT78D2 gene | This work |
| BS211G | BS211 derivate, overexpression of *Arabidopsis thaliana* UGT78D2 gene | This work |
| BS212G | BS212 derivate, overexpression of *Arabidopsis thaliana* UGT78D2 gene | This work |
| BS213G | BS213 derivate, overexpression of *Arabidopsis thaliana* UGT78D2 gene | This work |
| BS214G | BS214 derivate, overexpression of *Arabidopsis thaliana* UGT78D2 gene | This work |
| BS215 | BS215 derivate, overexpression of *Arabidopsis thaliana* UGT78D2 gene | This work |
| BS216G | BS216 derivate, overexpression of *Arabidopsis thaliana* UGT78D2 gene | This work |
| BS217G | BS217 derivate, overexpression of *Arabidopsis thaliana* UGT78D2 gene | This work |
| BS218G | BS218 derivate, overexpression of *Arabidopsis thaliana* UGT78D2 gene | This work |
| BS219G | BS219 derivate, overexpression of *Arabidopsis thaliana* UGT78D2 gene | This work |
| BS220G | BS220 derivate, overexpression of *Arabidopsis thaliana* UGT78D2 gene | This work |
| BS221G | BS221 derivate, overexpression of *Arabidopsis thaliana* UGT78D2 gene | This work |
| BS300G | BS300 derivate, overexpression of *Arabidopsis thaliana* UGT78D2 gene | This work |
| BS301G | BS301 derivate, overexpression of *Arabidopsis thaliana* UGT78D2 gene | This work |
| BS302G | BS302 derivate, overexpression of *Arabidopsis thaliana* UGT78D2 gene | This work |
| BS303G | BS303 derivate, overexpression of *Arabidopsis thaliana* UGT78D2 gene | This work |
| BS304G | BS304 derivate, overexpression of *Arabidopsis thaliana* UGT78D2 gene | This work |
| BS305G | BS305 derivate, overexpression of *Arabidopsis thaliana* UGT78D2 gene | This work |
| BS306G | BS306 derivate, overexpression of *Arabidopsis thaliana* UGT78D2 gene | This work |
| BS400G | BS400 derivate, overexpression of *Arabidopsis thaliana* UGT78D2 gene | This work |
| **Plasmids** |  |  |
| pP*_43_*NMK | Amp^R^, Kan^R^, *E. coli*-*B. subtilis* shuttle vector | [1] |
| p*P_43_*-UGT78D2 | p*P_43_*NMK derivate with UGT78D2 cloned | This work |
| p7Z6 | pMD18-T containing lox71-zeo-lox66 cassette | [2] |
| pTSC | Em^r^ Amp^r^; temperature sensitive in B. subtilis | [2] |

**Table S3** Primers used in this study.

| Primer | Sequence (5’-3’) |
| --- | --- |
| pgcA-L-F | ACGGAAAGACGAGATTTGGACAT |
| pgcA-L-R | ACGGTAGAATCTCGCACGTAAAAAGAAATAACGGGCT |
| pgcA-Z-F | TTCTTTTTACGTGCGAGATTCTACCGTTCGTATAGC |
| pgcA-Z-R | ACATACCACCTATCACTACCGTTCGTATAATGTATGC |
| pgcA-P43-F | TATACGAACGGTAGTGATAGGTGGTATGTTTTCGCTTGA |
| pgcA-P43-R | AAGTCATTTAATTCCTCCTCTTAAATACCGCTATCACTTTATATTTTACATAATCGC |
| pgcA-R-F | TAGCGGTATTTAAGAGGAGGAATTAAATGACTTGGAGAAAGAGCTATGAACG |
| pgcA-R-R | GATGTACAGAAATGGACGTCAGT |
| gtaB-L-F | TGTCATCTGTCGATTGAGCTCTT |
| gtaB-L-R | ACGGTAGAATCTCATGGTTAAACTATTCTCTATTTACCCTTTTCA |
| gtaB-Z-F | TAGAGAATAGTTTAACCATGAGATTCTACCGTTCGTATAGC |
| gtaB-Z-R | AAAAAAGACACCCTTCTACCGTTCGTATAATGTATGC |
| gtaB-PsrfA-F | ATACGAACGGTAGAAGGGTGTCTTTTTTTGCCTTTTTTTC |
| gtaB-PsrfA-R | TCATTTAATTCCTCCTCTTAAATACCCCTAATCTTTATAAGCAGTGAAC |
| gtaB-R-F | TAAAGATTAGGGGTATTTAAGAGGAGGAATTAAATGAAAAAAGTACGTAAAGCCATAATTCCA |
| gtaB-R-R | AAGATGGCAAGATTAGAAGGTGCT |
| pgi-L-F | CGATATCCAAAAATGCCGGATCAAT |
| pgi-L-R | TACGAACGGTAGAATCTCTAATGTGAGAAAGCTGACTGGCATT |
| pgi-Z-F | TCAGCTTTCTCACATTAGAGATTCTACCGTTCGTATAGC |
| pgi-Z-R | ATTATACCGTTATGGAGGGACTACCGTTCGTATAATGTATGC |
| pgi-R-F | ATACGAACGGTAGTCCCTCCATAACGGTATAATGTTTTCAT |
| pgi-R-R | AATGAACCCTGATTCCGTGATTACAAA |
| ydfO-L-F | TGGTCGGAAAGATCAGCGATCAA |
| ydfO-L-R | AACGGTAGAATCTCTAGCCATTTTTATCCGCTCCTTT |
| ydfO-Z-F | ATAAAAATGGCTAGAGATTCTACCGTTCGTATAGC |
| ydfO-Z-R | TACTTCAAACGGCACTACCGTTCGTATAATGTATGC |
| ydfO-R-F | ATACGAACGGTAGTGCCGTTTGAAGTAAGAGAGTTAGA |
| ydfO-R-R | CAGAACTGCGTGACATACTGTACA |
| mhqA-L-F | ATGACGCATAAAAAGAGAACTGCT |
| mhqA-L-R | AACGGTAGAATCTCATGCTGTCCCTCCAGGTTATTTTA |
| mhqA-Z-F | TGGAGGGACAGCATGAGATTCTACCGTTCGTATAGC |
| mhqA-Z-R | TCAATTTTGCTTCACTACCGTTCGTATAATGTATGC |
| mhqA-R-F | ATACGAACGGTAGTGAAGCAAAATTGAAACCGATCCAA |
| mhqA-R-R | CTCCAACAACGAAAAATGCTGCTAA |
| yodE-L-F | AGAGCAGTTCCAAAAGCAATTTGA |
| yodE-L-R | AACGGTAGAATCTCACCTGAACTTTAAAAGGGGGAATT |
| yodE-Z-F | TTAAAGTTCAGGTGAGATTCTACCGTTCGTATAGC |
| yodE-Z-R | AATAGGAGTGAGACTACCGTTCGTATAATGTATGC |
| yodE-R-F | ATACGAACGGTAGTCTCACTCCTATTTATCTCGATTTCAAGA |
| yodE-R-R | GAAGGCATATGTACGGAATAAGCAT |
| yetO-L-F | TTCTGGCATCGATTCTCCGTACT |
| yetO-L-R | AACGGTAGAATCTCTCGAACCATTCCTTTCCGTATGA |
| yetO-Z-F | AAGGAATGGTTCGAGAGATTCTACCGTTCGTATAGC |
| yetO-Z-R | TATCCCTGCCCAGACACTACCGTTCGTATAATGTATGC |
| yetO-R-F | ATACGAACGGTAGTGTCTGGGCAGGGATATAGATAAAGA |
| yetO-R-R | TGAAAATCCCGTCTCCCATGTTT |
| catE-L-F | ATGAAGCGACAAGCAACATTGAT |
| catE-L-R | AACGGTAGAATCTCATGCTGCATGCCTCTTTTCTTAT |
| catE-Z-F | AAGAGGCATGCAGCATGAGATTCTACCGTTCGTATAGC |
| catE-Z-R | TGTAATATAGGCGCCGGACTACCGTTCGTATAATGTATGC |
| catE-R-F | ATACGAACGGTAGTCCGGCGCCTATATTACATTTGT |
| catE-R-R | ATGCCTCCCTGAGATATGAAAGT |
| yetG-L-F | AGAAGTAGGTGCTATCAATATGGGAA |
| yetG-L-R | AACGGTAGAATCTCTGCTGTCAAACAAGGGACATACAA |
| yetG-Z-F | TTGTTTGACAGCAGAGATTCTACCGTTCGTATAGC |
| yetG-Z-R | TTCTTTGACATTGAGAATCTACCGTTCGTATAATGTATGC |
| yetG-R-F | ATACGAACGGTAGATTCTCAATGTCAAAGAAAGCGCA |
| yetG-R-R | TTCTCAAGGAGCCGTTTTGTTAGT |
| yhjG-L-F | TCATCGCCGTATTGGTGATCACT |
| yhjG-L-R | AACGGTAGAATCTCTTCACCACTCCTTTTGTTTCATGAA |
| yhjG-Z-F | ACAAAAGGAGTGGTGAAGAGATTCTACCGTTCGTATAGC |
| yhjG-Z-R | ACGAATCAATATGGTATGCTACCGTTCGTATAATGTATGC |
| yhjG-R-F | ATACGAACGGTAGCATACCATATTGATTCGTCCCGATG |
| yhjG-R-R | GGAAATCTGATTTACGTTACAGCCTT |
| moxC-L-F | ATTTCTGCTGCAAAACTGGGAAT |
| moxC-L-R | AACGGTAGAATCTCATGAATAAAATCTGCACGTGTCATCTT |
| moxC-Z-F | ACGTGCAGATTTTATTCATGAGATTCTACCGTTCGTATAGC |
| moxC-Z-R | AACCTAATGATGTGATGAATCTACCGTTCGTATAATGTATGC |
| moxC-R-F | ATACGAACGGTAGATTCATCACATCATTAGGTTTCGTCT |
| moxC-R-R | AGTCTGATATGGATAGGAGCCTTCTA |
| yoaI-L-F | CATTTTCCCATTGCCCTGTATTTCA |
| yoaI-L-R | AACGGTAGAATCTCCTCCTTCATATTATGCTGCGTATTGT |
| yoaI-Z-F | AGCATAATATGAAGGAGGAGATTCTACCGTTCGTATAGC |
| yoaI-Z-R | TGCTCCTGCTTTGGATAACTACCGTTCGTATAATGTATGC |
| yoaI-R-F | ATACGAACGGTAGTTATCCAAAGCAGGAGCAGTTGAA |
| yoaI-R-R | CTGGTTTGAGGAGGACCAAATATGA |
| ycnE-L-F | CGAGTCTGGTGAATATCAGAAGCA |
| ycnE-L-R | AACGGTAGAATCTCGTCGTTATGCGAATTTATTGTTCGAAT |
| ycnE-Z-F | TAAATTCGCATAACGACGAGATTCTACCGTTCGTATAGC |
| ycnE-Z-R | TACTCGCTAAGCTCTGTCTACCGTTCGTATAATGTATGC |
| ycnE-R-F | ATACGAACGGTAGACAGAGCTTAGCGAGTAAAGGAA |
| ycnE-R-R | AAAATCCGACAGGCCGAATAAGA |
| yxaG-L-F | AGAAGTAGTTGAGCGGATTATACGTAA |
| yxaG-L-R | AACGGTAGAATCTCATCGAAGCATTAGATTTAAAGGTGATGA |
| yxaG-Z-F | AAATCTAATGCTTCGATGAGATTCTACCGTTCGTATAGC |
| yxaG-Z-R | ACTGGTCTAAATTGGAGGACTACCGTTCGTATAATGTATGC |
| yxaG-R-F | ATACGAACGGTAGTCCTCCAATTTAGACCAGTCTATATATTATCA |
| yxaG-R-R | TGTCATTGAGAGATCCCTCCTAGA |
| bglH-L-F | TATGGAAGGATCCTGTTGCATCAAA |
| bglH-L-R | TATACGAACGGTAGAATCTCTATCAGCAGGTTATCGCCACAAA |
| bglH-Z-F | ATAACCTGCTGATAGAGATTCTACCGTTCGTATAGC |
| bglH-Z-R | AAACTAATGGGGTGATCAACTACCGTTCGTATAATGTATGC |
| bglH-R-F | TACGAACGGTAGTTGATCACCCCATTAGTTTTTTCAAGA |
| bglH-R-R | GCTCAAAACGGCCACGATTATTTA |
| bglC-L-F | AAAAAGACGGATCACAGGTCAGT |
| bglC-L-R | TATACGAACGGTAGAATCTCATTCTGGATGCTGGTGGATCATA |
| bglC-Z-F | AGCATCCAGAATGAGATTCTACCGTTCGTATAGC |
| bglC-Z-R | TTTGTCTTGATGACATCTCTACCGTTCGTATAATGTATGC |
| bglC-R-F | TTATACGAACGGTAGAGATGTCATCAAGACAAACGGAGA |
| bglC-R-R | ACGTCTCAAACGAAAGAATATGATGA |
| bglA-L-F | GTCTTGAAGAGCATGTGAAGGAATAT |
| bglA-L-R | TATACGAACGGTAGAATCTCAGGAACTATAAGAAGCGCTCGAAT |
| bglA-Z-F | TTCTTATAGTTCCTGAGATTCTACCGTTCGTATAGC |
| bglA-Z-R | TAAGCTGAGGAGGAATTACTACCGTTCGTATAATGTATGC |
| bglA-R-F | TATACGAACGGTAGTAATTCCTCCTCAGCTTACGACTGTA |
| bglA-R-R | TCGACAGAAGGCTGATATAGCCTT |
| UGT78D2-F | agcGGTACCTTAGGTAAGAGAGGAATGTACACATGACGAAACCGAGCGATCCTACA |
| UGT78D2-R | tagTCTAGATTAGATGATATTAACAACTGCATCAAGTAATCCT |

Underlined letters represent homologous sequence.

**Reference**

1. Zhang XZ, Cui ZL, Hong Q, Li SP: High-level expression and secretion of methyl parathion hydrolase in *Bacillus subtilis* WB800. *Appl Environ Microbiol.* 2005, 71**:**4101-4103. https://doi.org/10.1128/aem.71.7.4101-4103.2005

2. Yan X, Yu HJ, Hong Q, Li SP: Cre/lox system and PCR-based genome engineering in *Bacillus subtilis*. *Appl Environ Microbiol.* 2008, 74**:**5556-5562. https://doi.org/10.1128/AEM.01156-08
